# Supplementary material for: Implementation and enforcement of mandatory calorie labelling regulations for the out-of-home sector in England: Qualitative study of the experiences of business implementers and regulatory enforcers
Source: PLoS One. 2025 Jul 2;20(7):e0323452. doi: 10.1371/journal.pone.0323452 (PMC12221163; doi:10.1371/journal.pone.0323452)
Supplement: Attachment — Submitted filename: PONE-D-24-11664.docx [file pone.0323452.s005.docx]

**Title:** Implementation and enforcement of mandatory calorie labelling regulations for the out of-home sector in England: qualitative study of the experiences of business implementers and regulatory enforcers

**Manuscript no:** PONE-D-24-11664

**Overall Comments:**

The manuscript describes the importance of caloric labelling and its implementation in food outlets in England. Barriers and facilitators of this policy impacted in different way. I would like to acknowledge the authors for their valuable contributions in this manuscript. The topic is both timely and important, and the study offers interesting insights and makes its worth impact.

**Suggestions:**

**Abstract:** In the abstract it needs to clearly describe the purpose of the study. How sampling technique suits your methodology. I could not understand how many is your target population and how much is your sample size. What is the ratio between target population and sample size? Either your sample size is statically significant?

**Introduction:** The introduction should clearly state the problem being explored. This introduction highlights the importance of labelling policy but does not clearly describe the source i.e which institution or lab can do the test.

From Author point of view does labelling mean Nutrition value test of the product and its result?

Lack of clear research emphasis makes it less likely for the reader to understand the real purpose of the study. Please explain in detail.

**Data and methodology:** The authors have used the term Local Authorities (LAs) many times in the manuscript but I am unable to find what does it mean? Does the study include the customer or user of these products with their point of view about labelling formalities? What they are demanding?

**Results and Discussion:** The term “out of home food business” what is the context of this terminology?

What is the difference between caloric labelling and nutrition value labelling? What are the different aspect of theses discussed in this section?

As this is qualitative study so briefly discuss the theory which is in context of this study? How the study is supported by this theory? This section does not adequately compare the study findings to the existing literature.

**Conclusion Policy implication, limitation, and future recommendation:** Finally, summarize the key findings and clearly link them to the research objectives. Explain the limitations of the study and future recommendations, such as conducting comparative studies in the region and suggesting any variables for future researchers.

**Recommendation:** Paper needs **major revision** in the lights of the points mentioned above
